# Minds without language represent number through space: origins of the mental number line

**DOI:** 10.3389/fpsyg.2012.00466

**Published:** 2012-10-31

**Authors:** Maria Dolores de Hevia, Luisa Girelli, Viola Macchi Cassia

**Affiliations:** ^1^Laboratoire Psychologie de la Perception, Université Paris Descartes, Sorbonne Paris Cité, CNRS UMR 8158Paris, France; ^2^Cognitive Neuroimaging Unit, NeuroSpin, INSERM U992Saclay, France; ^3^Dipartimento di Psicologia, Università Milano-BicoccaMilano, Italy

During the last decades, extensive research has investigated both the developmental origins and the representational format of numerical information. A crucial contribution to these issues comes from recent studies on non-verbal populations, such as non-human animals and preverbal infants, which suggest that number is intuitively and fundamentally spatial in nature, that a predisposition to relate numerical information to spatial magnitude emerges very early in life, and that the association of numbers to different spatial positions critically depends on biologically determined processing and attentional biases.

Various sources of evidence suggest that when representing numbers human adults translate them into corresponding spatial extensions and positions (Restle, 1970; Galton, 1880; Dehaene et al., 1993; Fias et al., 1996). This phenomenon is referred to as number-space mapping and accounts for various systematic behavioral effects in numerical and visuo-spatial tasks. For instance, numerical processing modulates spatial representation according to a cognitive illusion, whereby small numbers induce a compression and large numbers an expansion of spatial extent (de Hevia et al., 2006, 2008; Stöttinger et al., 2012). In particular, adult's bisection of a line flanked by two numbers is biased toward the larger one (Fischer, 2001; de Hevia et al., 2006; Ranzini and Girelli, 2012), and the reproduction of a spatial extension is underestimated when delimited by two small numbers, and overestimated when delimited by two large numbers (de Hevia et al., 2008). Other observations, such as the interference between numerical and physical size in Stroop-like tasks and cross-dimensional mapping tasks (Stevens, 1970; Girelli et al., 2000; Pinel et al., 2004; de Hevia et al., 2012), support the existence of a mapping between symbolic and non-symbolic numbers and spatial magnitude.

The idea we would like to put forth is that the number-space mapping appears to be fundamental, spontaneous, and present very early in life, as it might constitute an innate trait of human, and possibly non-human, cognition. The notion that this mapping is universal and spontaneous is supported by neuroanatomical evidence showing that common parietal structures are engaged in both numerical and spatial tasks (Dehaene et al., 2003; Fias et al., 2003). Critically, electrophysiological studies have revealed that the posterior parietal cortex in primates, which includes quantity-selective neurons, contains accurate information about discrete (number of items) and continuous (length) quantity, with the same neurons coding for both non-symbolic number and spatial length (Tudusciuc and Nieder, 2007). Therefore, in line with the well-known ATOM (A Theory Of Magnitude) model proposed by Walsh (2003), numbers, as well as other magnitudes, might not be represented in isolation but spontaneously connected to space representation.

Further support for an intuitive and universal number-space mapping comes from research conducted with preschool children, preverbal infants, humans in remote cultures, and non-human animals, where a spontaneous mapping between number and space has been observed through a variety of experimental paradigms. When bisecting a line flanked by two different, non-symbolic numbers, 3–5-year-old children show a signature bias toward the larger number, just as adults do (de Hevia and Spelke, 2009; Girelli et al., 2009). Through the habituation paradigm, infants at 8 months of age transfer the discrimination of an ordered series of numbers to an ordered series of line lengths, and learn and productively use a rule that establishes a positive relationship between number and length, while failing to do so with an inverse relationship (de Hevia and Spelke, 2010; see also Lourenco and Longo, 2010). Using the number line task, which explicitly requires the mapping of number onto space (Siegler and Opfer, 2003), adults living in an Amazonian remote culture, with little or no education, resemble children's mappings with non-symbolic numbers (Dehaene et al., 2008). These findings suggest that the number-space mapping takes place well before formal education, preceding language, and symbolic knowledge acquisition. Moreover, among other mappings between continuous dimensions the number-space mapping seems to have a privileged status. When preschool children create cross-dimensional matches between different instances from the dimensions of number, line length, and level of brightness, they reliably perform mappings between number and length, and only partially between brightness and length, but fail to map number and brightness (de Hevia et al., 2012). Also in adults, number establishes a stronger overlap, at both functional and neural levels, with the dimension of space than with the dimension of brightness (Pinel et al., 2004; but see Cohen Kadosh et al., 2008).

An instantiation of the number-space mapping is that ordered numerical magnitudes are associated to different spatial positions along a horizontal continuum. The classical finding for this phenomenon is the Spatial-Numerical Association of Response Codes (SNARC) effect: generally speaking, small numbers are responded faster with the left hand and large numbers with the right hand, suggesting a compatibility effect between the left and right sides of one's own body and a left-to-right oriented numerical representation (Dehaene, 1992; Dehaene et al., 1993; Fias et al., 1996). This phenomenon has been extended to a variety of scenarios; among others, priming with a small or large number leads to shifts of attention toward the left or right sides of the space, respectively (Fischer et al., 2003). Critically, SNARC-like effects have been described for non-numerical ordinal series: adults react faster using the left hand to the presentation of the initial letters of the alphabet (Gevers et al., 2003), initial tones of a musical scale (Rusconi et al., 2006), initial (or past) events (Santiago et al., 2007), and initial elements in a list of unrelated words (Previtali et al., 2010), while they are faster using the right hand for the final elements of these series. Therefore, ordinal information in general, and not only number, triggers the use of an oriented spatial code. Moreover, the association of number with spatial positions is amply malleable, so that by simply varying the task requirements or setting, like conceiving numbers as depicted in a clock-face (Bächtold et al., 1998) or exposing bilingual participants to reading different languages (Shaki and Fischer, 2008), the association changes. This suggests that associating numbers to spatial positions results from a task-dependent individual's mental strategy to organize information (Fischer, 2006), an instance of the spatial coding of ordinal information in working memory (van Dijck and Fias, 2011).

Contrary to what commonly hypothesized, the origins of this mapping might not be exclusively culturally based. In favor of a culturally based position, the SNARC effect is modulated by reading direction: in Western cultures, small numbers are associated to the left and large numbers to the right side, while in cultures with right-to-left reading/writing direction the association is weaker (Dehaene et al., 1993) or reversed (Shaki et al., 2009). However, although early attempts to trace the SNARC effect in children described its emergence at 9 years of age (Berch et al., 1999), recent studies using non-symbolic number and non-chronometric tasks found it in 4-year-old children not formally introduced to reading system (van Galen and Reitsma, 2008; Patro and Haman, 2012). Moreover, the 3- and 4-year-olds who exhibit a consistent left-to-right bias in tasks such as subtraction and addition of tokens and counting objects (e.g., counting from the left and proceeding rightwards) are more proficient at basic numerical knowledge (Opfer et al., 2010). These studies suggest that, much before entering school, early cultural factors engendered by activities such as counting or “reading” illustrated books (McCrink et al., 2011) may determine the specific orientation of children's number-space mapping.

Far from denying the strong impact of cultural conventions on the number-space mapping, we see these forces as playing a modulating and refining role, not a fundamental one. Our idea is that the association of numbers onto spatial positions along a spatial magnitude might root in early biases present in the processing of magnitude information, whether numerical or spatial, which, from early on in development, would concur in shaping the way infants attend and represent any ordinal information, such as number. Optimal candidates might be a biologically determined advantage for processing the left hemispace, and an advantage in the processing of increasing order. Across the lifespan, these biases would be modulated and refined by exposure to cultural conventions.

In fact, and of critical importance to our view, not all processing biases are determined by culture. Let us review the seminal studies on counting abilities in newly hatched chicks. In these studies, chicks are trained to peck at the 4th position in a series of ten identical, equispaced and sagittally oriented locations. Afterwards, when required to identify the correct location within a new series identical to the one used at training, but horizontally oriented, chicks are more accurate at identifying the 4th position from the left than from the right end, which is chosen at chance level (Rugani et al., 2010). While cultural conventions cannot account for these findings, basic attentional biases can. The left bias shown by chicks is thought to be due to right hemispheric dominance in visuospatial processing, resulting in the left hemifield guiding the birds' behavior. Chicks' hemispheric lateralization can be experimentally manipulated by controlling the rearing environment of the eggs, thus providing a promising animal model for investigating the neural bases of the oriented number-space mapping (Vallortigara et al., 2010). This manipulation has been also performed in fish by obtaining animals that differ in the direction of cerebral lateralization. When these animals solve a bisection task, i.e., choosing the central element in a row, strong spatial biases are found in opposite directions, either toward the right or the left, depending on the artificially obtained direction of cerebral lateralization (Dadda et al., 2009).

These findings from non-human, non-linguistic species substantiate the role of neural factors and visuo-spatial processing strategies in engendering attentional biases. One contribution to the emergence of a number-space mapping in humans is, in our view, the biologically determined attentional bias regulating the asymmetrical exploration of space. Although available infant literature does not clearly establish the presence and degree of such biases, hints for this phenomenon are nonetheless informative. First, classical studies on infants' visual exploration indicate that at birth horizontal scans are wider and more frequent than vertical scans (Haith, 1980), suggesting that visual exploration and stimulus detection are easier along the horizontal than the vertical orientation. Second, a timing asymmetry may exist in the maturation of cerebral hemispheres, with a temporal advantage for the right over the left hemisphere (Rosen et al., 1987).

Thus, spatio-temporal constraints on brain development may determine an advantage of the left over the right visual hemispace in early infancy. This leftward spatial bias might constrain both the exploration of external space and the organization of information along a representational space. In fact, attentional biases in visual space likely extend to the mental representation of information. For instance, patients with unilateral neglect not only fail to explore the left side of visual space, but also the left side of a mental image (Bisiach and Luzzatti, 1978), and fail to accurately bisect imagined numerical intervals, showing biases toward the larger number (Zorzi et al., 2002; Vuilleumier et al., 2004). A further processing bias relevant to our argument is the recently disclosed advantage for processing increasing magnitude information. Four-month-old infants discriminate increasing ordered sequences of an object progressively changing in size, but fail at detecting decreasing sequences (Macchi Cassia et al., 2012). These finding points to the existence of an asymmetry in the processing of ordinal information which, combined with a natural propensity to asymmetrically explore space, might constitute one of the building blocks of a mental mapping where numbers are associated to different spatial positions. From early on and across the lifespan, the advantage in the horizontal scanning of the left hemispace, and the advantage in the processing of ascending order might combine with culturally based factors, such as exposure to reading/writing habits and the associated scanning and ordering routines. These factors would either counteract a predetermined orientation or strengthen it, eventually giving rise to culturally dependent strategies to represent ordinal information, including, but not limited to, number.
